# Untangling Inositol (Pyro)Phosphate Biology Through Emerging Technologies

**DOI:** 10.1002/advs.76711

**Published:** 2026-07-20

**Authors:** Adolfo Saiardi, Mengsi Lu, Yue Zhang, Anuj Shukla, An‐Li Andrea Ko, Henning J. Jessen

**Affiliations:** ^1^ Laboratory For Molecular and Cell Biology University College London London UK; ^2^ Institute of Organic Chemistry University of Freiburg Freiburg Germany; ^3^ CIBSS – Centre for Integrative Biological Signaling Studies University of Freiburg Freiburg Germany

**Keywords:** cell signaling, metabolism, phytic acid, stable isotopes

## Abstract

Inositol phosphates (InsPs) and inositol pyrophosphates (PP‐InsPs) constitute a remarkably diverse yet evolutionarily conserved signaling network generated through combinatorial phosphorylation of *myo*‐inositol. Despite their widespread presence across eukaryotes, progress in understanding their biology was long constrained by major analytical challenges arising from their extreme charge density, structural similarity, and isomer diversity. The advent of new analytical approaches is now transforming the field. High‐resolution analytical platforms, including capillary‐electrophoresis mass spectrometry, liquid‐chromatography mass spectrometry, and inductively coupled plasma‐based detection, enable sensitive quantification and isomer discrimination. Parallel developments in chemical synthesis, stable‐isotope labeling, and NMR spectroscopy provide essential reference standards and permit investigation of their metabolic turnover in living systems. These developments have uncovered unrecognized InsP and PP‐InsP isomers, revealed substantial variation in metabolic networks across organisms and cell types, and exposed unexpectedly dynamic turnover. Emerging biological insights highlight InsPs and PP‐InsPs as versatile cellular regulators that link metabolism to cellular signaling, influencing cellular energetics and phosphate homeostasis. These messengers are involved in diverse processes both in the cytoplasm and in the nucleus, controlling protein complex assembly and protein pyrophosphorylation. Together, these advances shed light on InsPs and PP‐InsPs as adaptable signaling molecules and open new avenues for deciphering their physiological roles in health and disease.

## Graphical abstract

Inositol (pyro)phosphates represent a vital class of intracellular messengers that govern multiple biological processes. The last decade has experienced a surge of interest in the functions of this class of signaling molecules, primarily due to the introduction of several innovative analytical technologies. A wide array of biological discoveries enabled by the new technologies is currently being investigated and critically assessed. This review seeks to summarize and discuss recent advancements and their wider implications for the field of research.

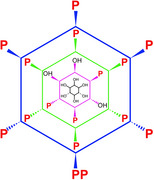

## 1 Introduction

The carbohydrate *myo*‐inositol (Ins) in its phosphorylated forms plays key roles in a multitude of signal transduction mechanisms. The discovery of Ins(1,4,5)P_3_ as a second messenger mobilizing calcium from intracellular stores [1] has prompted an explosion of interest in the InsP research area. Continued research revealed that this family of molecules is ubiquitously present in eukaryotic organisms [2]. Numerous InsPs coexist within cells, with the six‐times phosphorylated ring of InsP_6_ (inositol hexakisphosphate, also known as phytic acid) representing the predominant species, accompanied by the ever‐present, highly energetic messengers known as inositol pyrophosphates (PP‐InsPs) [3, 4]. Although the structural and metabolic complexity of the InsP and PP‐InsP network appears daunting, this very intricacy underscores its physiological significance. Nonetheless, research into InsP and PP‐InsP functions did not witness a progressive increase in interest. During the 1990s, attention shifted from the water‐soluble InsPs to their lipid‐anchored counterparts: the phosphoinositides (PtdInsPs). This inadvertent diversion of attention was fueled by PtdInsP's relative simplicity, given that only seven are known to exist, and by the development of fluorescent biosensors that enabled their imaging [5]. Nonetheless, the steady, continuous work of a few groups helped to lay an important foundation for a second explosion of interest in InsP and the exponential growth that the research field is currently witnessing. This review aims to evaluate these recent developments and their impact on the research area.

**FIGURE 1:**
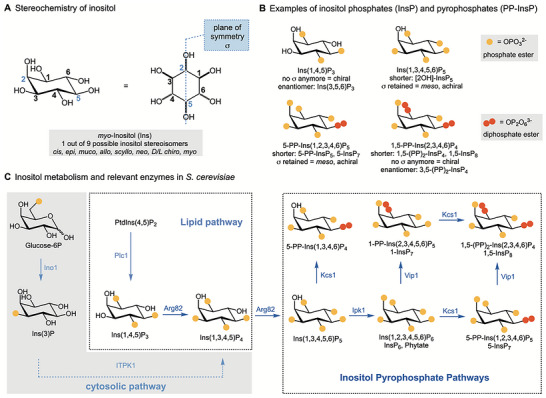
Overview of inositol structures, phosphorylation patterns and metabolism: (A) Stereochemistry of *myo*‐inositol (Ins) in different depictions with a plane of symmetry (σ) dissecting the 2 and 5 positions; Notice that substitution outside of the plane of symmetry generates enantiomers from prochiral precursors. (B) Examples of different common phosphorylations in InsPs and PP‐InsPs that can or cannot break the internal symmetry. Different abbreviations are indicated; (C) *Saccharomyces cerevisiae* synthesizes InsPs using the lipid pathway. This synthetic route is initiated with the hydrolysis of the phosphoinositide PtdIns(4,5)P_2_ by Plc1 (Phospholipase C), generating Ins(1,4,5)P_3_, metabolised by Arg82 (Inositol polyphosphate multikinase) to Ins(1,3,4,5,6)P_5_ that is then converted to InsP_6_ by Ipk1 (Inositol 1,3,4,5,6‐pentakisphosphate 2‐kinase). PP‐InsPs are synthesized by Kcs1 (inositol‐hexakisphosphate‐kinase) and Vip1 (Bifunctional inositol‐pyrophosphate‐kinase and phosphatase) diphosphorylating positions five and one, respectively. Expressing human ITPK1 (Inositol tetrakisphosphate 1‐kinase 1) in yeast creates the cytosolic pathway [6] normally absent in yeast [7]. This route begins with the conversion of glucose‐6P to Ins(3)P carried out by Ino1 (Inositol‐3‐phosphate synthase), followed by conversion to higher phosphorylated InsPs by ITPK1 without any phosphoinositide intermediate.

Before discussing how recent discoveries are shaping our knowledge of InsP and PP‐InsP metabolism and signaling, it is essential to outline the biochemical foundations of their synthesis and diversity. Inositol, owing to its metabolic stability [8], has been used by evolution as a framework for combinatorial phosphorylation that underpins today's InsP and PP‐InsP metabolic and signaling networks. There are nine inositol stereoisomers, several of which are found in nature [9]. This review focuses on the phosphorylated forms derived from *myo*‐inositol (inositol hereafter), as it constitutes over 95% of the known inositol pools in animals, plants, and yeasts [10]. Inositol can be generated in two steps from glucose‐6‐phosphate (G6P), which is first converted to inositol‐3‐phosphate (Ins(3)P) by inositol phosphate synthase (IPS or MIPS for *myo*‐inositol phosphate synthase; INO1 in yeast, ISYNA1 in mammals), and subsequently dephosphorylated by monophosphatases such as inositol monophosphatase 1 (IMPA1). Inositol carries six hydroxyl groups with a plane of symmetry bisecting the positions 2 and 5 (Figure 1A). Combinatorial phosphorylation can give rise to 63 different InsPs [11]. Moreover, numerous (if not all) carbon atoms can accommodate diphosphate groups, producing PP‐InsPs, further amplifying their diversity [12].

This complexity that hindered advancement of the field is gradually being addressed through a multitude of analytical innovations that have emerged over the last decade. Advances in separation‐based technologies have pushed analytical resolution and sensitivity to unprecedented limits. Complementary progress in chemical synthesis, isotope labeling, and enzymatic methods has yielded reference compounds critical for accurate quantification and isomer resolution [13]. An impressive range of discoveries is now under active exploration and critical evaluation. To contribute to this effort, we provide an analysis of the chemical and biological properties of InsPs and PP‐InsPs, highlighting how these molecular features have inspired and shaped recent analytical developments. We then evaluate how this progress is altering our understanding of the InsP and PP‐InsP metabolism and discuss how these small‐molecule messengers affect protein functions.

## 2 Emerging Analytical Technologies

The study of InsPs and PP‐InsPs has long been hindered by analytical challenges. These molecules are highly charged, lack chromophores, and exist in dozens of regio‐ and stereoisomeric forms. For decades, the field relied primarily on radioactive labeling coupled to strong anion exchange (SAX) chromatography and scintillation detection [12, 14, 15], or on indirect colorimetric assays [16]. These approaches provided sensitivity but offered limited isomer discrimination, posed safety concerns, and were constrained by availability. More recently, polyacrylamide gel electrophoresis (PAGE) of extracted PP‐InsPs [17] has made analytics much more accessible to the broader community, but analytical issues regarding specificity and sensitivity remain unresolved. To address these obstacles, the field has expanded into other chromatographic and radiolabeling approaches in the past decade [18], embracing high‑resolution separation‐based technologies coupled to mass spectrometry (MS), such as capillary electrophoresis (CE)‐MS [19, 20, 21, 22, 23, 24, 25, 26, 27] and high performance liquid chromatography (HPLC)‐MS [28, 29, 30, 31, 32, 33, 34, 35], and inductively coupled plasma (ICP)‐based methods [36, 37, 38]. In addition, separation‐free methods such as two‐dimensional nuclear magnetic resonance (2D NMR) detection and stable isotope‐labeling approaches are now applied [39, 40].

## 3 Separation‐Based Analytics

### 3.1 Capillary Electrophoresis‐Mass Spectrometry (CE‐MS)

CE‐MS is becoming an increasingly important technology for InsP and PP‐InsP analysis. First described in 1995 [41], it has undergone significant improvements since 2020 [19]. A key prerequisite for this development was the implementation of TiO_2_ enrichment into InsP‐extraction protocols [21, 22, 42]. Recently, CE‐MS combined with ^18^O‐water pulse‐labeling enabled direct monitoring of InsP and PP‐InsP turnover and fluxes, exploiting its femtomole sensitivity to quantify ^18^O‐labeled isotopologues [24]. The datasets collectively argue for a linear rather than cyclic mode of PP‐InsP turnover [43]. In addition, CE‐MS enabled the discovery of new PP‐InsP isomers (4/6‐InsP_7_, 2‐InsP_7_) in plants and mammals that had previously been misassigned to the 5‐InsP_7_ pool [21, 26, 27, 44, 45]. A plant root‐specific PP‐InsP isomer with six phosphates was also identified in *Arabidopsis* by CE‐MS; intriguingly, its levels rise during phosphate starvation, when all other inositol pyrophosphates decline [27]. Despite significant efforts, this isomer's molecular structure still remains unresolved [46]. This highlights a key limitation of CE‐MS: its dependence on authentic (ideally isotope‐labeled) often non‐commercial standards, since InsP‐separation and peak shape vary greatly with sample matrix and ionic strength. Nevertheless, the inherently low injection volume of CE (nL) minimizes sample and precious standard consumption, which to some extent mitigates this limitation.

### 3.2 Liquid Chromatography‐Mass Spectrometry (LC‐MS)

While CE‐MS offers excellent resolution of highly charged regioisomers, LC‐MS remains the most accessible technique, as such instrumentation is available in many laboratories. Initial developments for tandem MS (MS/MS) applications using ion‐exchange separations [33, 47] have triggered further innovation. Hydrophilic interaction liquid chromatography (HILIC), on the other hand, improved the separation of InsP_6_ and PP‐InsPs in complex matrices. In particular, adding methylenediphosphonic acid to the mobile phase reduced peak tailing by minimizing interactions between phosphate groups and metal components of the LC system [28, 29, 30]. References produced by stable isotope labeling cell culture (SILAC) have also been applied in HILIC workflows [34]. Extending the analytical repertoire further, ion‐exchange chromatography MS/MS has been applied to the analysis of InsP_2_ to InsP_8_ in the parasite *Trypanosoma cruzi* [32]. Using ^13^C‐glucose labeling, this method uncovered lipid‐independent InsP‐synthesis in *T. cruzi* through conversion of glucose‐6‐phosphate to Ins(3)P [31].

### 3.3 Inductively Coupled Plasma (ICP)‐Based Methods (IC‐ICP‐OES, LC‐ICP‐MS)

Beyond the structural information obtained from CE‐MS and LC‐MS, element‑selective detection enabled by ICP instruments provides a fundamentally different analytical capability. When analytes are introduced into an ICP, they are atomized and yield element‐specific ions, which can be detected using optical emission spectroscopy (OES) or MS. For InsPs and PP‐InsPs, phosphorus serves as the optimal detection element. InsP_6_ and its degradation products have been analyzed by ion‐pair LC‐ICP‐MS already in 2004 [48]. Recently, there has been a revival of such methods. Henninger et al. developed IC‐ICP‐OES to resolve 28 InsP isomers in 30 min in food matrices [37]. Carroll et al. pushed sensitivity further with LC‐ICP‐MS, achieving sub‐picomolar detection and mapping of soil InsPs using sample amounts as low as 1 mg [36]. LC‐ICP‐MS was also used to map InsP and PP‐InsP metabolism in avian tissues [38]. These recent developments are promising, as the complete atomization of analytes and matrices eliminates the need for enrichment steps and provides high sensitivity. However, care must be taken: It is essential to verify that peaks eluting into the plasma are well‐resolved from any co‐eluting phosphorus‐containing impurities. The potential presence of phosphorus‐containing contaminants, e.g., polyphosphate in yeast, might be problematic, as these cannot be distinguished once incinerated. Furthermore, as with CE‐MS, LC‐ICP‐MS instrumentation remains uncommon in most laboratories.

**TABLE 1:** Overview of different analytical platforms for InsP and PP‐InsP analyses.

Method	Advantages	Disadvantages	Analytes	LOD^*^ (S/N = 3)
**CE‐MS/MS**	InsP and isomer separation High sensitivity (fmol LODs) Low injection volume (10 nL)	Sample pre‐treatment Mobility time shift High salt sensitivity	InsP_2‐8_ PP‐InsP_4_ ^18^O‐InsP_2‐8_	0.45–1.5 fmol
**LC(HILIC/RP)‐MS/MS**	InsP separation High sensitivity (f‐pmol LODs) Stable migration time	Sample pre‐treatment Difficult isomer separation	InsP_1‐8_	1.9 fmol ^a^ −20 pmol
**LC‐ICP‐MS**	InsP and isomer separation good sensitivity (pmol LODs) No sample pre‐treatment Salt‐tolerant	Injection volume (10‐100 µL) Expensive instrument/limited access Complex and high maintenance	InsP_2‐8_ *myo*/*scyllo* isomers	3.3 pmol ^b^
**IC‐ICP‐OES**	InsP and isomer separation Affordable instrument	High injection volume (100 µL) Moderate sensitivity (p‐nmol)	InsP_2‐6_	34–200 pmol ^c^

* When pmol values were not explicitly provided, they were derived from the reported concentrations (µm or µg/mL) using the corresponding injection volume and the molecular weight of the neutral species. Limit of detection (LOD) range (based on reported values for all InsPs)

^a^ LOD values were derived from the lowest calibrated concentration and the corresponding injection volume reported.

^b^ LOD was back‐calculated from the reported LOQ using LOD = LOQ/3.

^c^ The analyte was detectable at the reported level, but no quantitative LOD/LOQ was provided in the original paper.

### 3.4 Separation‐Free Analytics

#### 3.4.1 Nuclear Magnetic Resonance (NMR) Assignments

Without relying on physical separation prior to detection, NMR enables direct, separation‑free interrogation of InsPs and PP‑InsPs. NMR is an information‐rich technology that however lacks the required sensitivity [47]. As the concentrations of InsPs and PP‐InsPs in cells can often be very low, ^31^P‐NMR has not been widely applied to study cell extracts, but instead, enriched samples [49, 50], enzymatic reactions [51, 52], and enantiomer assignments with chiral solvating agents [46, 53, 54]. Recently, the power of NMR to interrogate InsPs was reinforced by introducing fully ^13^C‐labeled inositol. This enables tracking of InsP and PP‐InsP metabolism in label‐fed cells by measuring extracts without any enrichment or separation, relying instead on 2D NMR fingerprinting using bilinear rotation decoupling (BIRD) ^1^H‐^13^C heteronuclear multiple‑quantum coherence (HMQC). Enantiomer assignment with asymmetrically ^13^C‐labeled inositol is now achievable, and has been applied to study phytate metabolism by the gut microbiota [55]. This is an area of great interest, since the agri‐biotech industry aims to enhance phytate assimilation by supplementing phytase in pig and broiler feed [56, 57, 58]. Additionally, ^13^C‐ NMR was instrumental to delineate the complex in‐cell activity of the mammalian multiple inositol polyphosphate phosphatase 1 (MINPP1) and its dephosphorylation network to produce Ins(2)P [40]. Moreover, conformational changes (the “ring‐flip”) in PP‐InsPs can be tracked [59]. These achievements warrant particular attention, since NMR remains the only approach that—if further developed—has the potential to resolve InsPs’ metabolism and enantiomers without analytical separation.

#### 3.4.2 Isotopic and Synthetic Reference Standards

**FIGURE 2:**
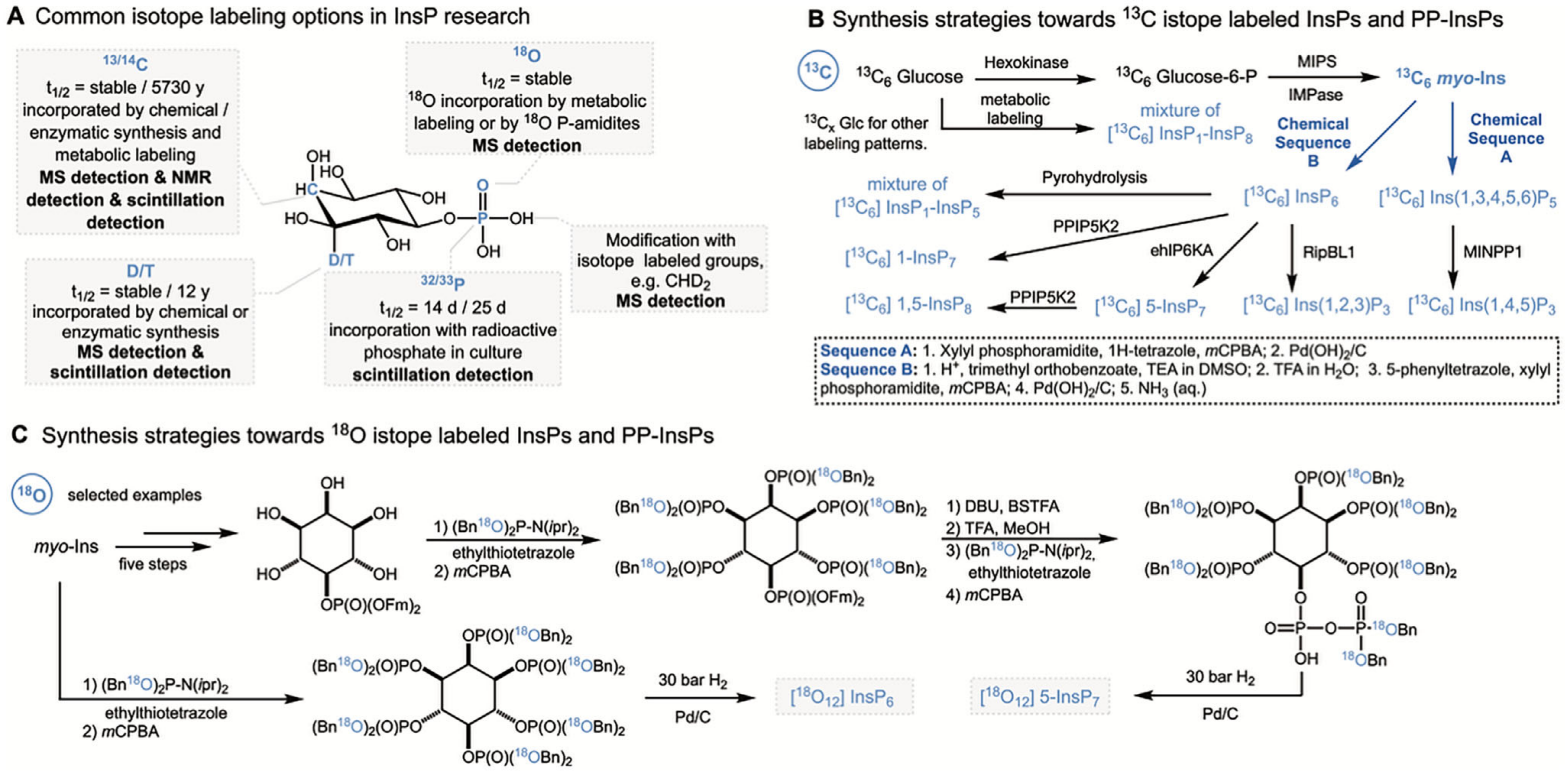
Overview of labeling strategies and selected examples of stable isotope‐labeled InsPs and PP‐InsPs accessed by chemical/chemoenzymatic syntheses. (A) Diverse isotope‐labeling strategies for InsPs are possible, including stable and radioactive isotopes facilitating different detection methods; (B) Different strategies targeting [^13^C]‐labeled InsP and PP‐InsP derivatives. MIPS (*myo*‐inositol‐3‐phosphate synthase or simply IPS); IMPase (inositolphosphate phosphatase); MINPP1 (multiple inositol polyphosphate phosphatase 1); PPIP5K2 (diphosphoinositol pentakisphosphate kinase 2); ehIP6KA (*Entamoeba histolytica* InsP_6_ kinase A); RipBL1 (*Ralstonia solanacearum* phytase). (C) Examples of chemical syntheses of [^18^O]‐labeled InsPs with late‐stage introduction of the labels. Using an ^18^O‐labeled P‐amidite, Ins can be directly transformed into benzyl‐protected ^18^O‐labeled InsP_6_ and then hydrogenated to give [^18^O_12_]InsP_6_ on a gram‐scale. The ^18^O‐labeling approach can also be applied to other (established) synthesis routes, e.g., as shown for the synthesis of [^18^O_12_]5‐InsP_7_. This enables the introduction of diverse combinations of labels in desired positions. (Bn^18^O)_2_P‐N(*i*pr)_2_ [^18^O_2_] Dibenzyl *N,N*‐di*iso*propylphosphoramidite; *m*CPBA meta‐chloroperoxybenzoic acid; DBU 1,8‐Diazabicyclo[5.4.0]undec‐7‐ene; BSTFA *N,O*‐Bis(trimethylsilyl)trifluoroacetamide; TFA Trifluoroacetic acid.

On the other hand, separation power alone is not enough. Without appropriate reference compounds, even the most sophisticated CE–MS or LC–MS workflows cannot provide confident assignment and accurate quantification. Stable isotope labeling, chemoenzymatic methods, and targeted synthesis have therefore become indispensable components of InsP and PP‑InsP analysis, enabling unambiguous isomer identification, calibration, and validation of emerging analytical workflows. These complementary tools, as summarized in Figure 2, form an essential foundation for both separation‑based and separation‑free methodologies.

#### 3.4.3 Deuterium (D) Labeling

Within the broader landscape of isotopic labeling methods, deuterium‑labeled inositols are emerging as an additional class of valuable references. A recent chemical synthesis has made 6‐times deuterated inositol accessible on a gram‐scale [60]. This building block has since been incorporated into PtdInsPs, and future syntheses will likely explore the utility of [D_6_]‐inositol for InsP research. Another approach to D‐incorporation has been pursued by Lämmerhofer et al. by introducing CHD_2_ groups via alkylation of phosphates in InsPs after extraction. This approach enables electrospray ionization positive mode (ESI^+^) measurements but cannot detect PP‐InsPs [35].

#### 3.4.4 Carbon‐13 (^13^C) Labeling

In addition to deuterium‑based labeling, substantial progress has been made in developing ^13^C‑labeled inositol and its derivatives. Harmel et al. developed a chemoenzymatic strategy to obtain [^13^C_6_]‐inositol through a three‐enzyme cascade from [^13^C_6_]‐glucose on a gram scale [39, 40]. Glucose is first converted to glucose‐6‐phosphate by hexokinase, then rearranged to Ins(3)P by MIPS and dephosphorylated by IMPase (or alkaline phosphatase; Figure 2B) [61]. Ramos‐Figueroa et al. followed a similar route to obtain all six possible inositol stereoisomers with a single ^13^C label in the ring [62]. The [^13^C]‐labeled inositols can then be used in metabolic studies through cell‐feeding, where they are converted to different phosphorylated forms traceable by NMR, CE‐MS, or HPLC‐MS [19, 39, 40, 55, 63]. Li and Lämmerhofer further extended this approach to a stable isotope labeling framework for LC‐MS quantification by direct feeding of [^13^C_6_]‐labeled glucose to an inositol‐independent cell line [34].

[^13^C_6_]‐labeled inositol can be readily converted chemically to [^13^C_6_]‐Ins(1,3,4,5,6)P_5_ or [^13^C_6_]‐InsP_6_ using P‐amidite chemistry. These compounds then serve as versatile precursors for further transformations, for instance, through selective dephosphorylation by phosphatases [22, 64], leading to [^13^C_6_]‐Ins(1,2,3)P_3_ and [^13^C_6_]‐Ins(1,4,5)P_3_, or via chemical pyrohydrolysis, giving heavy reference mixtures. Moreover, [^13^C_6_]‐labeled PP‐InsPs become accessible from [^13^C_6_]‐InsP_6_ using kinases such as IP6K from *Entamoeba histolytica* or PPIP5K from *Dictyostelium discoideum* [39, 63]. Collectively, these chemoenzymatic advances establish a robust platform for the scalable production of ^13^C‑labeled InsPs and PP‑InsPs, supporting metabolic tracing and absolute quantification across MS and NMR‐based analyses.

#### 3.4.5 Phosphate ^18^O Labeling (Chemical Synthesis)

A potential drawback of [^13^C_6_] labeling is that the isotope is introduced at the outset. Losses during synthesis are therefore expensive. To address this, a strategy that enables introduction of the isotope in the final steps thus offers clear advantages, in particular for lengthy chemical syntheses. Haas et al. developed a method for the chemical incorporation of ^18^O into phosphate groups via phosphoramidite chemistry [65]. Two examples are shown in Figure 2C. In the first, an [^18^O]‐labeled benzyl‐protected P‐amidite is used for the synthesis of [^18^O_12_]‐InsP_6_. The amidite is coupled to alcohols in the presence of ethylthiotetrazole (ETT), followed by oxidation with *m*CPBA and subsequent hydrogenolytic deprotection under high pressure. In the second step, the synthesis of 5‐InsP_7_ employs the same [^18^O]‐labeled P‐amidite. Here, the 5‐position is initially protected with an orthogonally removable fluorenylmethyl (Fm) phosphate‐group, which can be deprotected to allow installation of the β‐phosphate, which is also [^18^O]‐labeled. Final hydrogenation releases [^18^O_12_]‐5‐InsP_7_, which serves as a quantitative MS reference, despite partial ^18^O‐scrambling [23]. This chemistry was also applied to generate and assign the [^18^O_2_]‐4/6‐InsP_7_ isomer in mammalian tissues, as well as to synthesize different [^18^O_2_]‐PP‐InsP_4_ isomers [21, 46]. Altogether, these studies demonstrate the remarkable versatility of the ^18^O‐labeling approach: it can be integrated into existing synthetic routes, enabling rapid isotopic labeling.

### 3.5 Emerging Biology

#### 3.5.1 InsP and PP‐InsP Metabolism and Its Enzymology

Decoding of the InsP metabolism initiated immediately after the discovery of the Ca^2+^ release‐factor Ins(1,4,5)P_3_ [66]. Given its preeminent signaling role, research at the time mostly concentrated on its metabolism. This emphasis has skewed the comprehensive understanding of the InsPs metabolic network. In fact, the analytical progress made over the last decade is transforming our knowledge of InsPs metabolism. Only very recently, ^13^C‐NMR analyses have shown that Ins(1,2)P_2_ is the most abundant cellular inositol phosphate in diverse mamalian cell‐lines [40]. While Ins(1,2)P_2_ presence was recognised in mammalian cells before [67], the finding was not followed up, likely because it cannot directly originate from Ins(1,4,5)P_3_. A second phase of interpreting InsPs metabolism started with the cloning of the kinases phosphorylating the inositol ring, e.g., IP3‐3K and ITPK1 [68, 69]. Enzyme activity was assessed using recombinant enzymes to monitor in vitro InsP_x_‐to‐InsP_y_ conversion. The breakthrough came with the use of the budding yeast *Saccharomyces cerevisiae*, that enabled in vivo elucidation of the lipid‐dependent pathway from phospholipase C‐derived Ins(1,4,5)P_3_ to InsP_6_, 5‐InsP_7_, and ultimately 1,5‐InsP_8_ (Figure 1C; see also Table 2) [70, 71, 72, 73, 74]. Accumulation of non‐metabolized substrates in InsP‐kinases mutants, exemplified by Ins(1,4,5)P_3_ buildup in *arg82Δ* (Ipk2/IPMK; see Table 2), underscores how genetics and biochemistry work together to elucidate InsPs metabolism. Although budding yeast exhibits a simplified InsP metabolism and lacks a lipid‐independent pathway of InsP synthesis, this model organism has nevertheless been instrumental in elucidating the lipid‐independent cytosolic pathway of InsP_6_ synthesis [6] (Figure 1C), a pathway that is governed by ITPK1. Expression of human or plant ITPK1 in *plc1Δ* rescues InsP_6_ synthesis otherwise blocked at the level of PtdInsP_2_ [7]. The relevance of the cytosolic pathway to InsP_6_ and PP‐InsPs production differs across species. While in yeast the synthesis of InsP_6_ relies exclusively on PLC‐generated Ins(1,4,5)P_3_, the situation is different in *Trypanosoma brucei* and in *Dictyostellium discoideum*, where the synthesis of InsP_6_ depends entirely on the cytosolic pathway [31, 75]. It is likely that in mammals the cytosolic route plays a predominant role in generating InsP_6_.

**TABLE 2:** Overview of inositol phosphate kinases across species. See associate Supporting Table 1 in which the various accession numbers are reported.

**Inositol phosphate kinase Pfam entry**		**PF03770**				**PF17927** **PF05770**	**PF18086** PF00328	**PF06090**
**Lineage**	**Species Name**	**IPMK**	**IP3‐3K**	**IP6K**	**IPK**	**ITPK1**	**PPIP5K**	**IP5‐2K**
**Human kinase main activity**	**Main substrate**	Ins(1,4,5)P_3_	Ins(1,4,5)P_3_	InsP_6_		Ins(1,3,4)P_3_	5‐InsP_7_	2OH‐InsP_5_
	**Phosphorylating position(s)**	3 & 6	3	5		5 & 6	1	2
Metazoa	*Homo sapiens* (human)	IPMK	IP3KA IP3KB IP3KC	IP6K1 IP6K2 IP6K3		ITPK1	PPIP5K1 PPIP5K2	IPPK
	*Drosophila melanogaster* (fruit fly)	IPK2	IP3K1 IP3K2	IP6K		**—**	PPIP5K	IPPK
Amoebozoa	*Dictyostelium discoideum* (social amoeba)	IPMK	IP3‐3K	IP6K (I6KA)	IPKA IPKB	ITPK1	PPIP5K	IPK1
Fungi	*Saccharomyces cerevisiae* (baker's yeast)	ARG82 (IPK2)	**—**	KCS1		**—**	VIP1	IPK1
Euglenozoa	*Trypanosoma brucei brucei* (African trypanosomiasis parasite)	IPMK	**—**	IP6K		ITPK1	**—**	**—**
Viridiplantae	*Arabidopsis thaliana* (thale cress)	IPK2A IPK2B	**—**	**—**		ITPK1 ITPK2 ITPK3 ITPK4	VIH1 VIH2	IP5‐2K1* IP5‐2K2* IPK1
	*Triticum aestivum* (bread wheat)	IPMK1* IPMK2* IPMK3*	**—**	**—**		ITPK1* ITPK2* ITPK3* ITPK4* ITPK5* ITPK6* ITPK7* ITPK8* ITPK9* ITPK10* ITPK11* ITPK12* ITPK13* ITPK14* ITPK15* ITPK16* ITPK17* ITPK18*	VIH1* VIH2* VIH3* VIH4* VIH5* VIH6*	IP5‐2K1* IP5‐2K2*

* These kinases have been automatically annotated by UniProt prediction systems.

Structural insights now group the InsP‐kinases into four families (Table 2, Table S1). Many of these enzymes show substrate promiscuity; indeed, IPMK stands for Inositol Phosphate Multi‐Kinase, since it carries out the sequential phosphorylation of Ins(1,4,5)P_3_ to Ins(1,3,4,5,6)P_5_ [71, 72]. Additionally, evolution differentially amplified InsP‐kinase families in distinct eukaryote clades that have subsequently acquired analogous function [76]. Metazoa possess seven members of the PF03770 family. Besides IPMK, they host IP3Ks that metabolize Ins(1,4,5)P_3_ to Ins(1,3,4,5)P_4_, and the IP6Ks that convert InsP_6_ to 5‐InsP_7_. The viridiplantae (plants) *Arabidopsis thaliana* possesses two PF03770 family members of the IPMK‐type of enzymes (IPK2a/b). Conversely, the ITPK1 family expanded to four members, two of which convert InsP_6_ to 5‐InsP_7_ [49], functioning analogously to metazoan IP6Ks. The bread wheat *Triticum aestivum*, with its allohexaploid genome, witnessed an extraordinary amplification of the ITPK1 genes (Table 2). In wheat, these numerous ITPK1 genes are likely major drivers of its InsP metabolism.

The buildup of Ins(1,3,4,5,6)P_5_ (also referred to as [2‐OH]‐InsP_5_) in *ipk1*Δ yeast [70] has led to the prevailing notion that the biosynthesis of lower phosphorylated InsPs converges on [2‐OH]‐InsP_5_, with the phosphorylation of the 2‐OH group occurring last to produce InsP_6_. In mammalian HEK293T cells lacking the Ipk1 homologous IPPK (*ippk*‐/‐), InsP_4_ accumulates instead of [2‐OH]‐InsP_5_ [77]. InsP_4_ accumulation is also observable in *A. thaliana* atipk1‐1 mutant plants [78, 79]. These observations raise concerns regarding the overreliance on yeast as a model for validating the in vivo functions of InsP‐kinases from other organisms. The expression of human IPPK in *ipk1*Δ yeast can restore the levels of InsP_6_ [80], but its function in HEK293T cells is predominantly the phosphorylation of InsP_4_ rather than InsP_5_.

**FIGURE 3:**
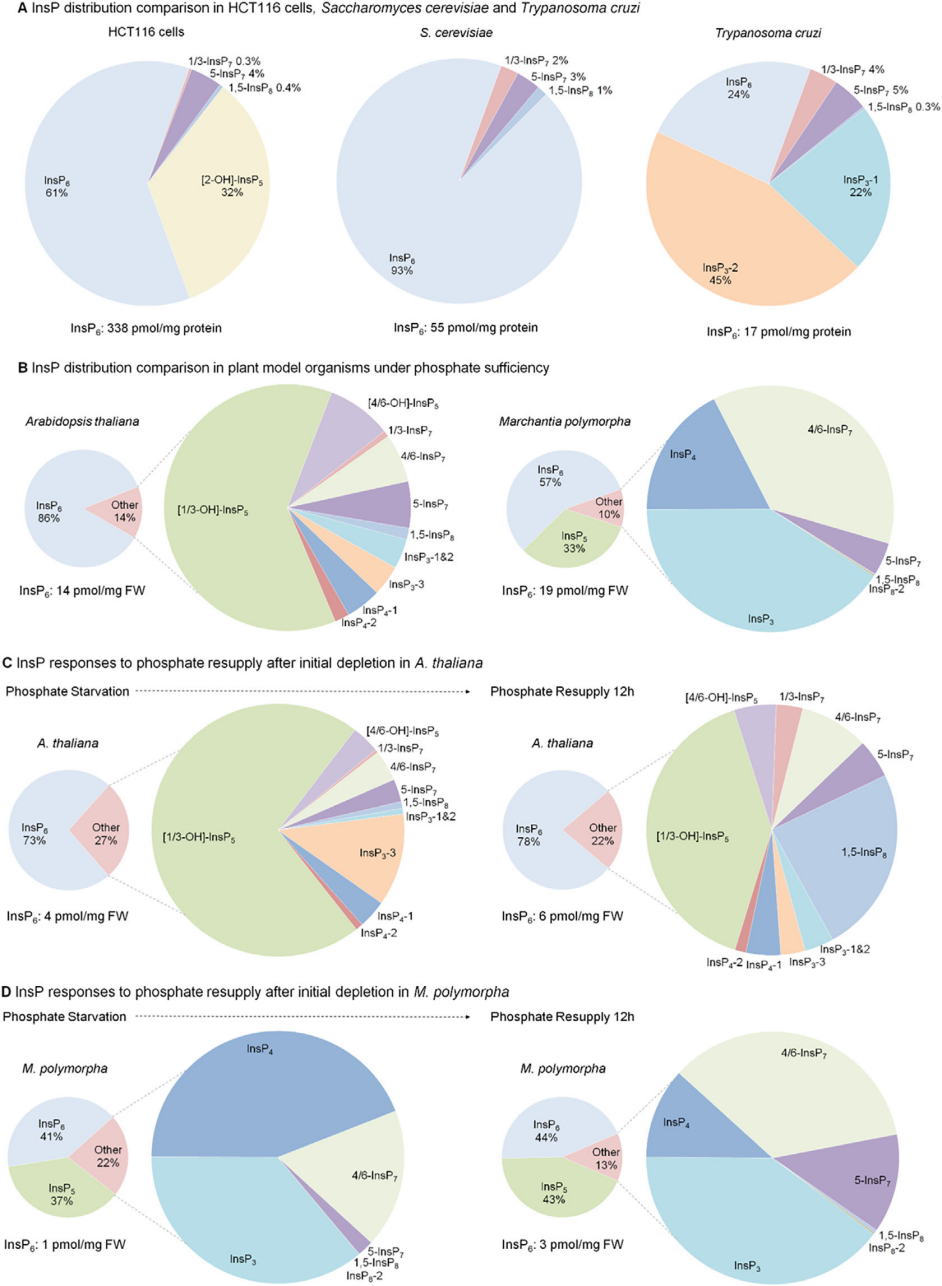
Overview of different InsP and PP‐InsP distributions across organisms measured by CE‐MS. (A) Main InsP and PP‐InsP detected in HCT‐116 cells (from InsP_5_ to InsP_8_), in *S. cerevisiae* (from InsP_6_ to InsP_8_), and in *T. cruzi* (from InsP_3_ to InsP_8_). InsP_6_ amount/mg protein is indicated, recalculated from the source publications [19, 20, 26, 27, 31, 32, 122, 132] to enable comparison (for yeast: 42 fL cell volume was assumed and 1 OD_600_ ≈ 1.3×10^7^ cells ≈ 0.2 mg protein; for *T. cruzi*: 10 pg protein per cell). Since some assumptions regarding OD, cell count, cell volume, etc. were made, these are approximated values. The identity of InsP3‐1 and InsP3‐2 has not been unambiguously established. (B) Comparison of InsP and PP‐InsP isomers in an angiosperm (*A. thaliana;* from InsP_3_ to InsP_8_) and a bryophyte (*M. polymorpha;* from InsP_3_ to InsP_8_) under phosphate‐sufficient conditions. Isomers that were not unambiguously assigned are labeled as e.g., InsP_3_‐1, InsP_4_‐1. InsP_3_‐1 and InsP_3_‐2 were not baseline separated and are reported as the sum of isomers. Absolute amount of InsP_6_ is indicated as pmol/mg of plant fresh weight (FW). (C) Responses of InsPs and PP‐InsPs when *A. thaliana* plants (grown in hydroponics) are shifted from P_i_‐deplete medium (left) to P_i_‐sufficient medium (resupply, 12 h after shift; right). Absolute InsP_6_ amount indicated as pmol/mg fresh weight (FW) of shoot tissue. **D** Responses of InsPs and PP‐InsPs when *M. polymorpha* Tak‐1 plants (grown in synthetic‐complete medium) are shifted from P_i_‐deplete medium (left) to P_i_‐sufficient medium (resupply, 12 h after shift; right). Absolute InsP_6_ amount indicated as pmol/mg fresh weight (FW) of thallus tissue.

**FIGURE 4:**
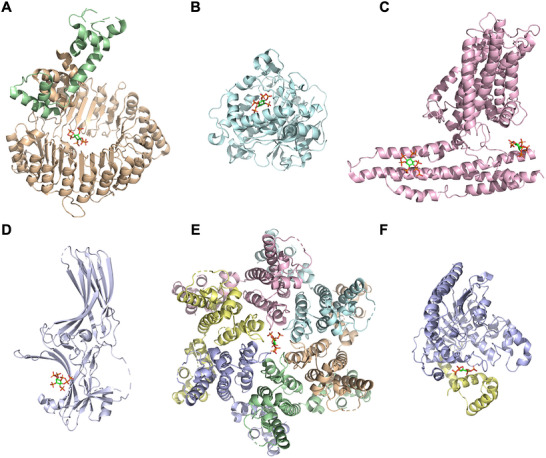
Representative structures of InsP‐binding proteins with their InsP ligands. (A) Crystal‐structure of the *Arabidopsis thaliana* TIR1‐ASK1 complex, with an InsP_6_ structural co‐factor (1.80 Å; PDB ID: 2P1M) [182]; (B) Crystal‐structure of the catalytic deaminase domain of human ADAR2, with its InsP_6_ co‐factor that is required for activity (1.70 Å; PDB ID: 1ZY7) [183]; (C) cryo‐EM structure of human XPR1, with two 1,5‐InsP_8_ molecules stabilizing its SPX domain (3.15 Å; PDB ID: 9JXH) [164]; (D) Crystal‐structure of the *Bos taurus* retinal Arr1, with a 5‐InsP_7_ co‐factor that could induce displacement of its C‐tail for activation (2.60 Å; PDB ID: 7MP0) [184]; (E) Crystal‐structure of the HIV‐capsid hexamer, with an InsP_6_ co‐factor that regulates capsid stability and uncoating through binding at its pore (2.00 Å; PDB ID: 6H09) [185]; (F) Crystal‐structure of the human HDAC3‐NCOR2^DAD^ co‐repressor complex, with an InsP_4_ co‐factor serving as intermolecular glue (2.06 Å; PDB ID: 4A69) [186].

The presence of InsP_8_ in trypanosomes [31] (Figure 3A) alongside the absence of PP‐IP5K [76] (Table 2) gene suggests that there is an undiscovered InsP‐kinase. Along these lines, in humans, twenty‐two PtdInsP‐kinases regulate six PtdInsPs [5], whereas only eleven InsP‐kinases currently appear to control over forty InsPs and PP‐InsPs [76]. This imbalance calls for initiatives aimed at discovering additional InsP and PP‐InsP kinases, or for a reassessment of their signaling properties. The notion of signaling promiscuity, originally describing how few ligands can trigger diverse receptor responses [81], contrasts with the complexity of the InsP and PP‐InsP metabolism. It generates many ligands and an even larger set of protein targets is available (for examples, see Figure 4) [82]. Such diversity raises questions about how signaling specificity is maintained.

Among InsP phosphatases, the inositol monophosphatases ‐particularly human IMPA1‐ are noteworthy because IMPA1 is the primary target of lithium‐ions (Li^+^). The uncompetitive inhibition of IMPA1 by Li^+^ [83] is crucial for understanding its therapeutic effect, mediated through inositol depletion [10, 84]. Another important InsP‐phosphatase is Multiple Inositol‐Polyphosphate‐Phosphatase 1 (MINPP1) that is mainly recognised as InsP_6_‐phosphatase of the phytase family. Human MINPP1 mutations are linked to pontocerebellar hypoplasia, a fatal neurodevelopmental disorder [85, 86, 87]. In vivo ^13^C‐NMR analyses have demonstrated that MINPP1 possesses a broad substrate specificity, culminating in the formation of Ins(1,2)P_2_ [40].

Given the diverse array of InsPs present in various cycling pools [24], it is unlikely that a handful of currently recognised InsP‐phosphatases (Table 3, Table S2) govern this intricate metabolism. As with the InsP kinases, it is likely that additional InsP‐phosphatases can be discovered. New analytical approaches should be employed to facilitate such discoveries and to determine whether e.g. lipid PtdInsP‐phosphatases influence InsP metabolism. The PtdIns(3,4,5)P_3_ phosphatase PTEN (phosphatase and tensin homolog) acts on Ins(1,3,4,5,6)P_5_ [88]. This discovery was not yet followed up by the community. Likewise, Lowe syndrome mutated protein OCRL (INPP5F), SHIP1 (INPP5D) and SHIP2 (INPPL1) all dephosphorylate Ins(1,3,4,5)P_4_ in vitro [89, 90, 91] but are considered exclusive PtdInsP phosphatases in vivo. In this context, it was recently shown that OCRL is inhibited by 5‐InsP_7_ [92].

**TABLE 3:** Overview of inositol phosphate phosphatases across species. See associate Supporting Table 2 in which the various accession numbers are reported.

**Inositol phosphate phosphatase Pfam entry**		**PF00459**	**PF22669**	**PF10409**	**PF00328**	**PF00328** PF18086	**PF00293**	**PF03162**
**Lineage**	**Species Name**	**IMPA** **INPP1**	**INPP5**	**PTEN**	**MINPP**	**PPIP5K**	**DIPP**	**SIW14**
Metazoa	*Homo sapiens* (human)	IMPA1 IMPA2 INPP1 2 putative	INPP5A 9 putative	PTEN 8 putative	MINPP1 5 putative	PPIP5K1 PPIP5K2	NUDT3 NUDT4 NUDT4B NUDT10 NUDT11 15 putative	**—**
	*Drosophila melanogaster* (fruit fly)	IMPA1* IMPA2* IMPA3* IMPA4* IMPA5* IMPA6* IMPA7* IPP 1 putative	INPP5* 5 putative	PTEN 1 putative	MIPP1 MIPP2 6 putative	PPIP5K	APS 9 putative	**—**
Amoebozoa	*Dictyostelium discoideum* (social amoeba)	IMPA1 2 putative	INPP5* 3 putative	PTEN1* PTEN2* PTEN3* 3 putative	MIPP1 4 putative	**—**	15 putative	DIPP* 3 putative
Fungi	*Saccharomyces cerevisiae* (baker's yeast)	INM1* INM2* 1 putative	4 putative	**—**	5 putative	VIP1	DPP1 5 putative	SIW14 4 putative
Euglenozoa	*Trypanosoma brucei brucei* (African trypanosomiasis parasite)	IMPA1* IMPA2* 1 putative	INPP* 3 putative	**—**	3 putative	**—**	4 putative	DIPP* 3 putative
Viridiplantae	*Arabidopsis thaliana* (thale cress)	IMPL1 IMPL2 VTC4 6 putative	15 putative	PTEN1 5 putative	MINPP*	VIH1 VIH2	29 putative	DSP1 DSP2 DSP3 DSP4 DSP5
	*Triticum aestivum* (bread wheat)	IMPA1* IMPA2* IMPA3* IMPA4* IMPA5* IMPA6* HisP1^#^* HisP2^#^* HisP3^#^* 14 putative	54 putative	19 putative	MINPP1* MINPP2* MINPP3*	VIH1* VIH2* VIH3* VIH4* VIH5* VIH6*	53 putative	DIPP1* DIPP2* DIPP3* DIPP4* DIPP5* DIPP6* 1 putative

*These phosphatases have been automatically annotated by UniProt prediction systems. The shared base names and numberings are here only to provide a reference framework.

^#^ HisP = Histidinol‐Phosphatase.

The phosphatases that act on the diphosphate in PP‐InsPs are becoming more thoroughly characterized. The first enzyme was found within the Nudix (nucleoside diphosphate linked to X) hydrolase family, which includes yeast Ddp1 and human Nudt3/4/10/11 [93]. These enzymes are often promiscuous: both yeast Ddp1 and human Nudt3 (also referred to as Dipp1) can metabolize PP‐InsPs, dinucleoside polyphosphates, and inorganic polyphosphate [94, 95, 96, 97]. Yeast Siw14 [98] and its plant orthologs, the Atypical Dual Specificity Phosphatases (PFA‐DSP1‐5) [99], constitute another category of phosphatases (absent in metazoa) that target the diphosphate. The most remarkable proteins acting on PP‐InsPs are the bifunctional kinases/phosphatases PPIP5Ks (budding yeast Vip1 and fission yeast Asp1). These proteins feature an ATP‐grasp kinase domain that is responsible for synthesizing the diphosphate at position 1 [100], alongside a phosphatase domain that specifically acts on the PP‐InsPs generated by the kinase [101]. Studies in fission yeast demonstrate that the phosphatase domain plays an equally important role as the kinase domain in maintaining balanced PP‐InsP levels [102]. In *S. pombe* Asp1, Aps1 (homologous to DDP1), and Siw14 work synergistically to prevent toxicosis resulting from the accumulation of PP‐InsPs [103]. While the deletion of individual pyrophosphatases leads to only slight increases in PP‐InsP levels, dramatic defects are observed in double mutants such as asp1/aps1 or aps1/siw14 [104].

#### 3.5.2 InsPs and PP‐InsPs Cellular Levels and Turnover

The discovery that cholinergic stimulation of pancreatic slices results in the incorporation of ^32^P_i_ into lipids [105] culminated in the identification of the GPCR‐PLC‐Ins(1,4,5)P_3_‐Ca^2+^ signaling paradigm [106]. This pivotal discovery (reviewed by Irvine [1]) sparked a significant surge of interest in the metabolism of InsPs, which was initially investigated using radioactive tracers, specifically ^32^P‐*ortho*phosphate to follow the movement of ^32^P among various InsPs. Subsequently, ^3^H‐inositol was applied with great success. This remarkable period of radioactive tracing utilized a variety of experimental models, including avian erythrocytes [107, 108, 109], amoebae [110, 111], mammalian cells [112, 113, 114], and plants [115, 116, 117].

The release of Ins(1,4,5)P_3_ occurs within seconds following GPCR stimulation, leading to a several‐fold increase in its concentration. During this same interval, an increase in Ins(1,4)P_2_ and InsP can also be observed, as Ins(1,4,5)P_3_ is rapidly dephosphorylated [118]. The velocity and magnitude of these changes are defining features of second‐messenger signaling molecules. With the exception of Ins(1,3,4,5)P_4_, which may see an increase in concentration after 30 min of GPCR stimulation [2, 119], all other InsPs are not directly influenced by GPCR activation. Consequently, a limitation in comprehending the functions of other InsPs is the absence of a swift stimulus‐dependent alteration. Additionally, fundamental to the understanding of the signaling of InsPs and PP‐InsPs is their dual role: they function both as transient metabolites in the synthesis pathways of other InsP species and as effector molecules in their own right.

The abundant InsP_5_ and InsP_6_ have been described as lethargic due to their slow metabolism [120]. Against this backdrop, the InsP_6_‐derived PP‐InsPs were identified. Their rapid turnover was particularly noteworthy. The application of sodium fluoride (NaF) demonstrated that in mammalian cells nearly 50% of the substantial InsP_6_ reservoir is transformed into derived PP‐InsPs every hour [3, 121]. This rapid turnover has amplified interest in PP‐InsPs. Regrettably, the physiological relevance remains elusive even at present. Fluoride inhibits the Nudix‐family of hydrolases, e.g., Dipp1 [93], yet it is a general phosphatase inhibitor with various effects on signaling. The recent application of ^18^O‐water labeling, elucidating in vivo phosphate group dynamics, validated the elevated turnover rate of PP‐InsPs in both yeast and HCT116 cells without NaF treatment [24, 43]. Intriguingly, the use of ^18^O‐water labeling on the amoeba *Dictyostelium discoideum* did not indicate high turnover of PP‐InsPs; rather, it suggested that InsP_6_ rapidly exchanges phosphate groups instead [24]. Differences of InsP and PP‐InsP metabolism between organisms are an emerging theme revealed by the recent advancement of InsPs analytics. These investigations are uncovering the differences in both the absolute concentrations and isomeric characteristics of InsPs and PP‐InsPs among diverse organisms (Figure 3A). Within the same taxonomic kingdom, the distribution of InsP‐ and PP‐InsP‐species can vary significantly, as exemplified by *Arabidopsis thaliana* [26] and *Marchantia polymorpha* [122] (Figure 3B). These distributions are also responsive to the growth medium [122, 123]. Furthermore, even distinct cell types within a single organism may exhibit distinct variations in InsPs; for instance, parallel CE‐MS analyses of mammalian cell lines have shown that PC3 and MCF7 cells contain higher levels of [2‐OH]‐InsP_5_ compared to InsP_6_, whereas InsP_6_ is predominant in other cell lines [19]. Another example is InsP_8_: in HCT‐116 cells (Figure 3A) cultivated for a long time in different laboratories, its levels diverge by an order of magnitude [20, 124].

The absence of rapid stimulus‐dependent changes on the second‐timescale in most InsPs and PP‐InsPs, despite their observed high turnover rates, has prompted a re‐evaluation of the function of PP‐InsPs as traditional second‐messengers [125]. This led to the hypothesis that these molecules may act as “metabolic messengers” [126], capable of detecting fluctuations in the metabolic state of a cell or organism. This emphasis was sparked by the IP6K's high K_m_, which falls within the 1.0–1.5 mm range for ATP [72, 127]. Consequently, variations in cellular energy levels could theoretically regulate PP‐InsPs synthesis. It is important to acknowledge, however, that numerous other enzymes also exhibit a high K_m_ for ATP. The primary integrator of metabolic signals, mTOR (mechanistic target of rapamycin), just like the IP6Ks, has a K_m_ for ATP that is slightly above 1 mM, leading to its initial characterization as a homeostatic ATP sensor [128]. The idea that mTOR functions as a direct ATP sensor has since been outmoded; mTOR actually senses cellular energy through AMPK and monitors nutrient availability via specialized amino acid sensors [129, 130]. This example should serve as a cautionary tale against the enthusiastic interpretation of the IP6Ks' high affinity for ATP as a justification for the role of PP‐InsPs as metabolic messengers and instead should encourage further research to elucidate the connection between basic metabolism and PP‐InsPs.

A recurring theme is the capacity of PP‐InsPs to monitor fundamental metabolic processes, particularly the availability of phosphate [94, 131, 132, 133, 134, 135, 136, 137]. The alterations in PP‐InsP levels in response to phosphate deprivation and subsequent replenishment occur over several minutes to hours. Given that phosphate (P_i_) is a crucial nutrient for plants [138], it is not unexpected that in plants one observes the most significant changes in PP‐InsP metabolism 12 h (and even more significantly 48 h) after phosphate resupply following prior deprivation (Figure 3C) [44, 122]. In *A. thaliana* 1,5‐InsP_8_ levels increase >100 folds in this time interval and also 1/3‐InsP_7_ changes dramatically. And while in *M. polymorpha* the relative changes in 1,5‐InsP_8_ are also large, one has to acknowledge that 5‐InsP_7_ and 4/6‐InsP_7_ appear to be main players at least in total abundance (Figure 3C). A common feature in both plants is the expansion of the overall InsP pool upon P_i_‐resupply (indicated by the increase in overall InsP_6_ abundance). It is important to highlight that the rise in PP‐InsPs after phosphate resupply following a period of starvation does not, in itself, dictate how plants respond to phosphate starvation. Nevertheless, the variations in PP‐InsPs relative to phosphate availability represent a primary area of research aimed at clarifying their physiological functions, which we will discuss next.

#### 3.5.3 InsPs and PP‐InsPs Regulated Functions

The cloning of InsP‐kinases enabled the generation of knockout models in yeast, amoeba, and later in mice and plants. These models linked the absence of specific InsP‐kinases—and the resulting loss of InsPs or PP‐InsPs—to distinct phenotypes. Notably, deletion of a single InsP‐kinase often produces multiple, diverse phenotypic effects. For example, in budding yeast, the almost complete removal of PP‐InsPs in *kcs1*Δ affects telomere length, vesicular trafficking, causes vacuole fragmentation, influences inorganic polyphosphate (polyP) synthesis, impacts bioenergetics, and helps maintain cell wall integrity [139]. This remarkable range of functions is also demonstrated by analysing phenotypes of InsP‐kinase‐KO mice [140]. Recent reviews have comprehensively covered these phenotypes [12, 141, 142, 143, 144]. Here, we focus on phosphate homeostasis regulated by PP‐InsPs [145], a process that may ultimately account, albeit indirectly, for the phenotypes observed in KO models.

#### 3.5.4 The InsP‐SPX Domain Communication

A conceptual shift occurred with the understanding that the SPX domain (Syg1/Pho81/XPR1) acts as a receptor for InsPs, particularly PP‐InsPs [146]. SPX is found in various yeast and plant proteins that regulate different aspects of P_i_‐homeostasis, ranging from transcription factors that manage P_i_‐responses to P_i_‐transporters [147, 148]. PP‐InsPs were linked to yeast P_i_ homeostatic mechanisms [131], such as the regulation of polyP‐synthesis [94, 149] and oversight of the PHO‐regulon [150]. This established a ligand‐SPX domain interaction, wherein the cellular P_i_‐status, conveyed through PP‐InsPs, is interpreted by proteins containing the SPX domain [151, 152].

Still, many aspects of this paradigm require better understanding. A genetic study indicated that in budding yeast, Kcs1 controls polyP‐synthesis with its product 5‐InsP_7_ binding to and regulating the SPX‐domains of the Vacuolar Transport Chaperone (VTC) complex, even if Vip1‐generated 1,5‐InsP_8_ has higher biding affinity to the SPX‐domain [146, 153]. Notably, in *Schizosaccharomyces pombe*, polyP synthesis is regulated by Asp1, a homolog of Vip1 and not by Kcs1 as in budding yeast [154]. Genetic studies have indicated that the Vip1 product 1‐InsP_7_ regulates the PHO‐regulon [150]. The current model posits that the PP‐InsPs bind to the SPX‐domain of the cyclin‐dependent‐kinase (CDK) inhibitor Pho81, which results in the transcription factor Pho4 being suppressed by the Pho80‐Pho85 kinase complex, leading to retention in the cytosol. By analysing PP‐InsP‐levels during P_i_‐starvation and resupply alongside PHO regulon activity, Chabert et al. proposed that 1,5‐InsP_8_ acts as the main regulator of the PHO‐regulon [132]. Some questions still remain: Kcs1, for example, is also necessary to synthesize 1,5‐InsP_8_ but *kcs1*Δ responds to P_i_‐starvation. Additionally, the O'Shea group has demonstrated that the minimal domain (MD) located towards the C‐terminus of Pho81 is crucial for translating the PP‐InsP signal. It is thus likely that both the SPX‐domain and the MD play a role in the regulation of PHO80‐PHO85 by PHO81 [155, 156]. These yeast findings have sparked broad interest in PP‐InsP‐mediated P_i_‐regulation in other organisms, particularly in plants [147] but also in humans.

XPR1 is the sole human protein that contains an SPX domain [157]. Initially, XPR1 was identified as the receptor for xenotropic/polytropic murine leukemia virus, leaving its physiological function ambiguous [158]. The later discovery of XPR1 as the first metazoan P_i_ exporter shifted its significance from a virological perspective to a key element of mammalian P_i_ physiology [159]. Insights from human genetics refined this physiological role: pathogenic variants of XPR1 were associated with primary familial brain calcification, and alleles derived from patients were found to hinder P_i_ efflux, thereby linking transporter function to disease manifestation [160]. The capability of cryo‐EM to structurally elucidate transmembrane proteins, along with the significance of XPR1 in human pathophysiology, has resulted over the past year in the publication of ten distinct structures [161, 162, 163, 164, 165, 166, 167, 168, 169, 170]. These studies, beginning with the characterization of the overall XPR1 architecture [167], have subsequently provided insights into the mechanisms of PP‐InsPs ligand‐gating, including the identification of two binding sites for PP‐InsPs [163, 169]. Collectively, these works have strengthened the concept of the ligand‐gated cycle, wherein the binding of PP‐InsP stabilizes SPX and facilitates the opening of the intracellular gate. Furthermore, cryo‐EM has shed light on the XPR1‐KIDINS220‐protein complex, which was originally recognized as a vulnerability in ovarian cancer regulating the localization of XPR1 [171, 172]. The interaction of KIDINS220 with XPR1 constrains the SPX‐domain, inhibiting the opening of the intracellular gate unless 1,5‐InsP_8_ is present, which alleviates this constraint [164].

XPR1 demonstrates how the identification of a conserved ligand‐binding domain (SPX) along with its small‐molecule ligands (PP‐InsPs) can enhance mechanistic insight. Nevertheless, also in this example, numerous questions are still unanswered. In Drosophila, XPR1 exhibited significant intracellular localization [173], and even in human cells, XPR1 shows organelle localization [174]. These investigations highlight that both localization and protein interactions are equally crucial as SPX‐ligand affinity in determining the physiological role of XPR1.

Plants contain numerous proteins with SPX‐domains [157], and given that P_i_ is a crucial yet limited nutrient, the elucidation of the role of InsPs and/or PP‐InsPs in the P_i_‐sensing mechanisms of plants is especially important [138, 147]. In plants, the transcription factor PHR1 (Phosphate Response 1) serves as the primary regulator of the P_i_‐starvation response. Although PHR1 does not have an SPX‐domain, its activity is negatively influenced by a group of stand‐alone SPX‐domain‐containing proteins [145], such as SPX1. By interacting with SPX1, PP‐InsPs modulate the interactions between SPX1 and PHR1, thereby regulating the expression of the P_i_‐starvation‐response responsive genes [135]. Recently, the capability of SPX1 to directly interact with DNA has been demonstrated, leading to the proposal that PP‐InsPs might regulate this protein‐nucleic acid interaction instead of the SPX1‐PHR1 complex [175].

Nevertheless, the ability of PP‐InsPs to regulate plant phosphate homeostasis is corroborated by the phenotype of the *Arabidopsis vih1 vih2* double mutant, which is unable to synthesize 1,5‐InsP_8_ and shows a constitutive activation of P_i_‐starvation‐induced genes [137]. However, recent studies highlight that the functional relationship between PP‐InsPs and plant P_i_‐homeostasis is not always straightforward. *Arabidopsis* mutants ipk1‐1 and itpk1, deficient in the synthesis of InsP_6_ and 5‐InsP_7_, display altered P_i_‐homeostasis [27, 79]. Conversely, the Itpk4 mutant, which also results in a significant reduction of InsP_6_ and PP‐InsPs, does not exhibit similar P_i_‐related phenotypes [176]. Furthermore, the recent development of plants overexpressing the VIH1 Kinase‐Domain (designated as VIP2KD) that exhibit elevated levels of PP‐InsPs, paradoxically show an increase in the expression of genes responsive to P_i_‐starvation [177]. This observation aligns with the phenotype of the mrp5 null plant, which accumulates PP‐InsPs while maintaining an unchanged P_i_‐starvation response [27]. Mrp5 (ABCC5) is a member of the ATP‐binding cassette (ABC) family facilitating the translocation of cytosolic InsP_6_ into the vacuole [178, 179, 180]. Consequently, the relationship between PP‐InsP levels and P_i_‐homeostasis is more complex than suggested by the simple interaction between SPX‐domains and InsPs.

### 3.6 Structural Insights

Beyond XPR1, many proteins have been crystallized with InsPs or PP‐InsPs. A Protein Data Bank search (https://www.rcsb.org/) yields hundreds of such structures. Therefore, we must broaden our exploration of the proteins and protein domains that interact with InsPs and PP‐InsPs with the goal to identify rules of InsP‐protein coordination and directions to dissect InsP physiology. While detailed discussion of individual cases is beyond our scope, we emphasize features that can guide interpretation of InsP‐protein structures (Figure 4). In this context, InsP and PP‐InsP isomer‐specific pull‐down probes have been developed and applied to mammalian and plant cell extracts. The hitlists of potential PP‐InsP interacting proteins (interactomes) are available and can be screened for proteins of interest that might bind to different P‐P‐InsP [82, 181].

Unlike *E. coli*, insect cells possess an InsP metabolism. Protein expression in insect cells can consequently lead to detection of InsPs in resolved structures. For example, InsP_6_ was detected within the RNA‐editing enzyme ADAR2 [183], and [3‐OH] InsP_5_ and InsP_6_ were found in the plant jasmonate and auxin receptor complexes, respectively [182, 187]. Ins(1,4,5,6)P_4_ crystallized in the HDAC3‐co‐repressor complex [186]. This raises the question of whether it corresponds to the natural ligand or is instead a consequence of the distinct InsP metabolism in insect cells. In addition to these few examples, most protein‐InsP structures, including XPR1‐InsP_6/7/8_, were obtained by adding excess InsPs during the crystallization process. InsPs may remain undetected when randomly distributed in a crystal, but clusters of basic residues could concentrate them sufficiently for structural observation. Unexpectedly observed InsPs should prompt functional investigation, whereas InsPs originating from additives require interpretation in light of their abundance and synthesis rates. Also, the context surrounding the protein of interest should be considered. For example, visual arrestin (Arr1) was crystallised in the presence of added Ins(1,4,5)P_3_, InsP_6_, 5‐InsP_7_, and 1,5‐InsP_8_ and non‐hydrolysable analogues [184]. These InsPs crystallise in a similar fashion and similarly are priming but not activating Arr1. Understanding the physiological ligand should take into account the InsPs relative concentration and affinity to Arr1, but also the timescale of arrestin engaging with phosphorylated rhodopsin [188]. Thus, to identify the Arr1 physiological effector, we should ask if cGMP, generated by activated rhodopsin, rapidly alters distinct InsPs and/or PP‐InsPs pools. It is thus important to have detailed analytical information about InsPs and PP‐InsPs abundance and metabolism across species, demonstrating again the impact of the new analytical approaches highlighted in this review.

The InsP‐protein crystal structures archived in the PDB reveal at least three distinct functions. 1) Allosteric modulation: InsPs or PP‐InsPs can bind allosterically, inducing conformational changes that activate or inhibit enzymes or modulate the gating of transmembrane channels [169]. 2) Molecular glue: in the HDAC3 – Ins(1,4,5,6)P_4 –_ NCOR2 complex, InsP_4_ sits at the interface between HDAC3 and its corepressor, effectively acting as an intermolecular adhesive that creates an optimal interaction surface [186]. 3) Structural organizer: In mature HIV‐Gag hexamers, InsP_6_ binds two lysine rings and coordinates six Gag peptides, stabilizing the immature lattice. By positioning these peptides correctly, InsP_6_ organizes the overall Gag scaffold [189, 190]. However, the analysis of all these structures does not reveal any consensus in primary amino acid sequence or 3D‐folding of domains that we could generally define as InsP or PP‐InsP binding modules.

#### 3.6.1 The Implication of the Anhydride Moiety/ies of PP‐InsPs

While ATP can function allosterically ‐a classical example is the negative regulation of phosphofructokinase‐ [191] the majority of ATP is hydrolysed to provide energy or to transfer phosphate groups, thereby conveying information to other biomolecules. In a manner akin to ATP, the potential of PP‐InsPs to drive phosphate‐transfer reactions was promptly acknowledged [50]. Employing radiolabeled ^32^Pβ‐InsP_7_ it was discovered that the ^32^Pβ‐phosphate is transferred non‐enzymatically to a pre‐phosphorylated serine, resulting in the formation of pyro‐phosphoproteins [192, 193, 194]. Although the concept of protein pyro‐phosphorylation is exciting, the necessity to synthesize ^32^Pβ‐InsP_7_ has restricted the investigation of this posttranslational modification (PTM) [195, 196, 197].

This constraint is being lifted due to the development of a tandem‐mass‐spectrometry (MS/MS) approach that takes advantage of the unique fragmentations of pyro‐phosphorylated peptides. Application of electron‐transfer/higher‐energy collision dissociation (EThcD) enables identification of pyro‐phosphorylated peptides [198, 199]. The integration of this MS/MS procedure into a workflow for extraction and enrichment of pyro‐phosphoproteins has recently uncovered the presence of this PTM in human cells [200]. Notably, the primary proteins subject to pyro‐phosphorylation in vivo are the same proteins identified employing ^32^Pβ‐InsP_7_: nucleolin, NOLC1 and TCOF1, homologous to yeast NSR1 and SRP40 [192, 193]. Although the qualitative nature of the current workflow does not permit an assessment of the pervasiveness of this PTM within the human proteome, the undeniable presence of pyro‐phosphorylated proteins in cells should encourage further investigation.

### 3.7 Perspectives

Twenty‐five years after the seminal review “Back in the water: the return of the inositol phosphates” [2], the study of InsPs and PP‐InsPs has made remarkable progress. Yet, as we now realize, these waters truly run deep. The advent of revolutionary technologies has transformed InsP analytics from a purely descriptive field into a systematic, quantitative discipline. This progress is not just facilitating InsP and PP‐InsP discoveries but is also revealing previously unforeseen layers of intricacy. Recent advances have not yet been fully integrated into our understanding of the metabolic and signaling networks of InsPs and PP‐InsPs. As this integration progresses, we expect transformative insights to emerge. Nevertheless, challenges remain.

The expanding catalog of biological functions linked to each InsP and PP‐InsP, while certainly an exciting feature of the research field, also introduces unprecedented complexity and raises many questions. For example, are the numerous proteins crystallized in the presence of InsP_6_ bona fide receptors? The steady and abundant InsP_6_ may not influence protein function in the same manner as other small‐molecule messengers such as Ins(1,4,5)P_3_ or cyclic adenosine monophosphate (cAMP), which undergo rapid concentration increases within seconds following receptor activation. With the flurry of new analytical technologies now available, we should persist in looking for stimuli that induce rapid changes of specific InsP and/or PP‐InsP isomers, such that we can properly define them as second messengers. At the same time, we should consider alternative functional roles. Can InsPs and PP‐InsPs contribute to protein folding by organizing the three‐dimensional architectures of specific protein domains? Can they act as an intermolecular glue that stabilizes binding interfaces between protein complexes, as in the case of Ins(1,4,5,6)P_4_ that mediates the interaction between histone deacetylase 3 (HDAC3) and its co‐repressor NCoR [186]. To what extent are InsPs sequestered within proteins? Are we analyzing tightly protein‐bound InsPs, or are we overlooking this pool due to our extraction methods? For example, the detection of InsP_6_ in HIV‐1 Gag was only possible after denaturing the protein complex by boiling [185], an approach disruptive for PP‐InsP analysis. Future research must address these fundamental questions.

The current emphasis is on investigating specific InsPs without taking into account that their removal from cells, such as through the knockout of their synthesizing kinases, exerts pleiotropic effects on the entire InsP metabolic pathway. For instance, knocking out the mammalian IPMK not only depletes or significantly reduces the final product of the kinase, Ins(1,3,4,5,6)P_5_, but also perturbs InsP_6_ and the overall metabolism of PP‐InsPs. Similarly, the deletion of ITPK1 in plants not only diminishes 5‐InsP_7_ but also dramatically alters the levels of InsP_4_ [27, 79]. Therefore, isomer‐restricted approaches should be avoided; future studies should aim to analyze InsP and PP‐InsP metabolism in its entirety and ideally dynamic nature using ^18^O‐water pulse‐labeling [24].

The ostensibly static InsP and PP‐InsP picture we currently have contrasts with the extraordinarily dynamic cellular environment. It will be both intriguing and essential to investigate in greater detail the extent to which these small‐molecule messengers assemble into distinct combinations across various cell types and even within compartments of a single cell. The available InsP and PP‐InsP analytics depend on extraction following cell lysis, resulting in the loss of crucial spatiotemporal information that only microscopy‐based technologies can offer. This represents perhaps the most significant limitation currently faced by the field. Developing genetically encodable or chemical biosensors would therefore be a highly desirable development. While designing fluorescent probes that respond selectively to specific InsPs and/or PP‐InsPs in cells, and discriminate against the abundant InsP_6_ is challenging [201, 202], any effort in this direction is valuable. The “visualization” of InsPs could tackle the long‐standing issue regarding the subcellular distribution of various InsP species or InsP cycling pools [24]. Are InsPs and PP‐InsPs freely diffusible within the cytosol, as suggested by the InsP_6_/Mg^2+^(5) complex [203], or is controlled synthesis/degradation necessary to manage local concentrations?

Finally, although not elaborated upon in this review, numerous human diseases have been linked to changes in InsP metabolism [12, 143, 204]. While inhibitors targeting InsP kinases are currently in development [136, 205, 206, 207, 208], they have yet to advance to clinical trials. Reflecting on the tumultuous journey that PI3K inhibitors have faced over the past thirty years [206, 209], it is evident that a more profound comprehension of the InsP and PP‐InsP metabolic networks is crucial, not only for the precise identification of appropriate disease targets and the anticipation of potential toxicity and side effects, but also for minimizing the overall expenses related to translational research. The rapid advances in InsP research over the last decade have set the stage for transformative breakthroughs and the potential emergence of innovative therapeutic interventions. “*Aqua profunda est quieta*”—still waters run deep—yet the depths of InsP research are anything but quiet; they are churning with the excitement of new discoveries.

## Author Contributions

**Anuj Shukla**: Writing – original draft, Writing – review and editing. **Adolfo Saiardi**: conceptualization, funding acquisition, writing – original draft, writing – review and editing, visualization, supervision. **Yue Zhang**: writing – original draft, writing – review and editing. **Mengsi Lu**: writing – original draft, writing – review and editing. **Henning J. Jessen**: supervision, writing – review and editing, writing – original draft, funding acquisition, conceptualization, visualization. **An‐Li Andrea Ko**: writing – original draft, writing – review and editing, visualization.

## Conflicts of Interest

The authors declare no conflicts of interest.

## Supporting information




**Supporting File 1**: advs76711‐sup‐0001‐SupplementryTable.xlsx.

**Supporting File 2**: advs76711‐sup‐0002‐SupplementryTable.xlsx.

## Data Availability

The data that supports the findings of this study are available in the supplementary material of this article.
